# Assessing the efficiency and microbial diversity of H_2_S-removing biotrickling filters at various pH conditions

**DOI:** 10.1186/s12934-024-02427-9

**Published:** 2024-05-28

**Authors:** Abbas Abbas Rouhollahi, Minoo Giyahchi, Seyed Mohammad Mehdi Dastgheib, Hamid Moghimi

**Affiliations:** 1 Department of Microbiology, School of Biology, College of Science, University of Tehran, Tehran, Iran; 2 Microbiology and Biotechnology Research Group, Research Institute of Petroleum Industry, Tehran, Iran

**Keywords:** Biofilm, Biodesulfurization, Biotrickling filter, Hydrogen sulfide, Microbial biofilm

## Abstract

This study aimed to investigate the operation of three parallel biotrickling filters (BTFs) in removing H_2_S at different pH conditions (haloalkaliphilic, neutrophilic, and acidophilic) and their associated microbial population in the biodesulfurization process. BTF columns were inoculated with enriched inoculum and experiments were performed by gradually reducing Empty Bed Retention Time (EBRT) and increasing inlet concentration in which the maximum removal efficiency and maximum elimination capacity in EBRT 60 s reached their maximum level in haloalkaline condition (91% and 179.5 g S-H_2_S m^−3^ h^−1^). For visualizing the attached microbial biofilms on pall rings, Scanning Electron Microscopy (SEM) was used and microbial community structure analysis by NGS showed that the most abundant phyla in haBTF, nBTF, and aBTF belong to *Gammaproteobacteria*, *Betaproteobacteria*, and *Acidithiobacillia*, respectively. Shannon and Simpson indexes evaluation showed a lower diversity of bacteria in the aBTF reactor than that of nBTF and haBTF and beta analysis indicated a different composition of bacteria in haBTF compared to the other two filters. These results indicated that the proper performance of BTF under haloalkaliphilic conditions is the most effective way for H_2_S removal from air pollutants of different industries.

**Graphical Abstract:**

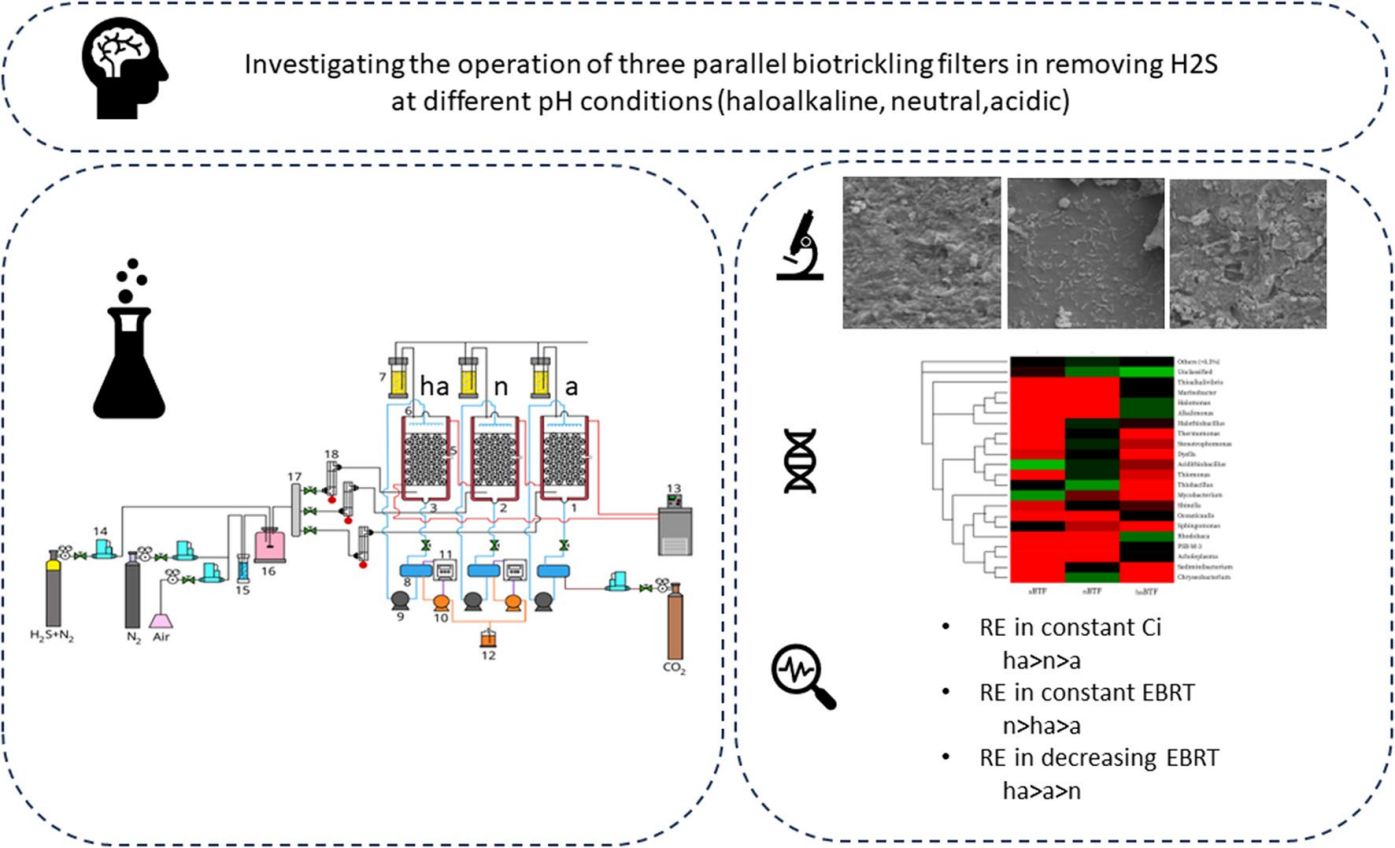

**Supplementary Information:**

The online version contains supplementary material available at 10.1186/s12934-024-02427-9.

## Introduction

Many industrial activities such as wastewater treatment, petrochemical and natural gas refineries, biogas, composting, pulp and paper, and food production discharge a significant amount of volatile sulfur compounds (VSCs) to the environment [1, 2]. The simplest VSC, hydrogen sulfide (H_2_S), is a colourless, flammable, and potentially explosive, heavier than air, highly poisonous and lethal at 600 ppm, severely corrosive, odour nuisance with rotten egg smell, and a very low odour threshold value [1, 3]. Fossil fuels, the main energy source around the world, contain sulfur compounds that generate oxidated sulfur mixtures during the burning process. These compounds are precursors of acid rain that destroy the natural environments and architecture [4, 5]. Hence, sulfur removal from fuels is necessary and sometimes challenging.

Massive research has been carried out in the field of removing H_2_S from gaseous fuels and off-gases, but still, new innovative research and new technologies are required to improve the performance of biological technologies and to elucidate the role of microbial communities in their functionality [6–9].

Traditional physicochemical technologies have been employed for the treatment of gaseous emissions containing VSCs of waste and/or sour gas streams. However, these methods in contrast to biological methods (biodesulfurization) [2, 10], which are based on the oxidation of H_2_S by sulfide oxidizing bacteria (SOB), are not very environmentally friendly and cost-effective due to high energy consumption for operating at high pressure and temperature [6, 11].

Biodesulfurization processes of gas streams are usually carried out by four types of conventional bioreactors: biofilters, bioscrubbers, biotrickling filters (BTF), and bubble column bioreactors [12–14]. Although all of them can effectively remove hydrogen sulfide from contaminated gaseous streams, BTFs have some advantages over others. In BTF aqueous nutrient phase is trickled over the packing bed and the gas phase is forced upward through the vessel. The contaminants are absorbed and biodegraded by the microbes growing on the packing surfaces as biofilm. This technology has been scaled up at industrial scales for the removal of up to 12,000 ppm H_2_S concentrations [15] and unlike bioscrubber and bubble column bioreactors, the processes of absorption, biodegradation of contaminating compounds, and regeneration occur simultaneously in a single column. In comparison to biofilters, BTFs are more effective in removing compounds that recalcitrant to degradation or generate acidic by-products [16–18] and environmental conditions such as humidity, temperature, pH, and discharge of toxic substances can be better controlled. The choice of the type of bioreactor depends on the nature and loading rate of contaminated gases [19–21].

In BTFs, the autotrophic Sulfide oxidizing bacteria (SOB) have the primary role in the detoxification of VSCs. Thus, the activity and composition of autotrophic and heterotrophic microbial communities significantly impact the efficiency and removal rate. On the other hand, environmental factors such as pH have a basic role both in the diversity of the microbial community and solubility of sulfidic compounds.

The effects of pH as a critical factor in the formation of bacterial community structures have been emphasized by various researchers including Tu et al. [22], Omri [23], and Chouari [24]. At different pH, varying autotrophic and heterotrophic microbial groups become active and dominant [25]. The selective pressure imposed by environmental conditions such as pH and concentrations of pollutants drastically changes the population structure and its diversity and ultimately affects system performance [26]. The results of Tu's 2017 research also showed that the biofilm thickness and stability are greatly affected by the inlet loading rate and pH [27].

This work aimed to investigate the effect of three different pH-adjusted conditions on the performance and microbial community in three parallel biodesulfurizing BTFs working in the same operational condition. For robust long-term operation, a new method of periodic relative starvation was used to control reactor clogging. Moreover, to elucidate the effect of pH on H_2_S utilizing bacterial community, the bacterial community composition of BTFs' attached biofilm was studied by next-generation sequencing (NGS) of the 16S rRNA gene. The simultaneous investigation of three BTFs in the same conditions except for pH and the proof of the differences in the consortia population promoting biological removal is the subject that has been less studied in depth.

## Results and discussions

### BTFs performance in experimental conditions

**Fig. 1:**
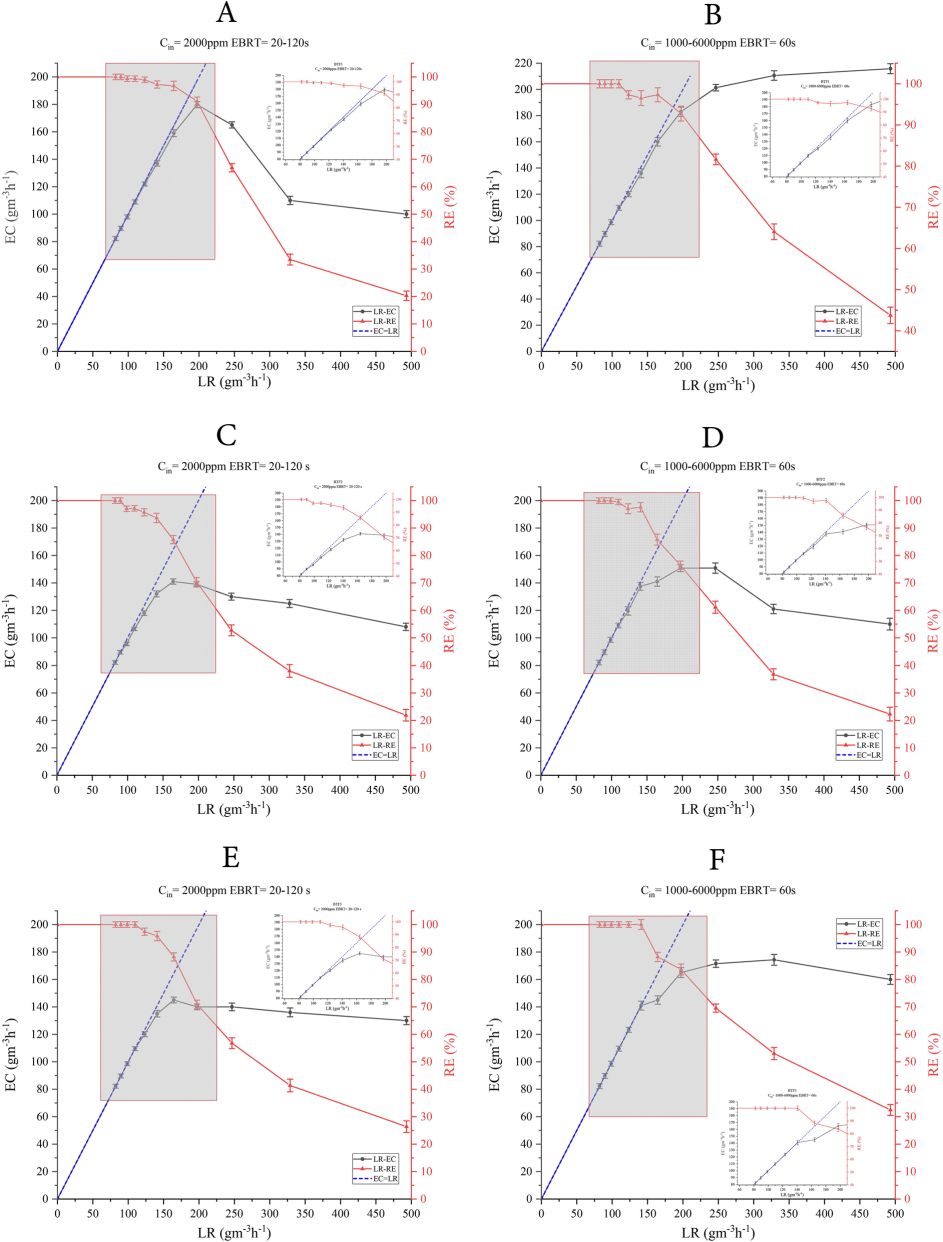
Effect of BTFs fed load on H_2_S elimination capacities during the treatment of H_2_S. haBTF (**A, B**), nBTF (**C**, **D**), aBTF (**E, F**). Constant inlet concentration (2000 ppm), EBRT Change from 120 to 20 s (**A, C**, **E**). Constant EBRT 60 s, inlet concentration changes from 1000 to 6000 ppm (**B, D, F**)

The results of the E1 experiment (Fig. 1) indicate that haBTF is more efficient in removing H_2_S than nBTF and aBTF. In this experiment, haBTF reached its maximum EC of 179.8 g m^−3^ h^−1^ in less EBRT (50 s) with 91.14% EC, which is 5.44% and 2.94% more efficient than nBTF and aBTF, respectively. The critical EC in which 97% of inlet H_2_S is removed by the systems was also determined to be 31.1 g m^−3^ h^−1^ and 17.5 g m^−3^ h^−1^ higher than nBTF and aBTF's EC, respectively.

In the E2 experiment (Fig. 1), the three filters were operated at constant EBRT (60 s), but with rising C_i_ (from 1000 to 6000 ppm). The results were similar to the E1 experiment, with haBTF reaching the maximum EC of 210.6 g m^−3^ h^−1^, which was higher than nBTF and aBTF by 59.8 g m^−3^ h^−1^ and 36.3 g m^−3^ h^−1^, respectively. The highest critical EC (167.8 g m^-3^ h^-1^) was also detected in haBTF. Although haBTF obtained the highest EC rate in the E2 experiment, the RE of the system was recorded at its highest point (91.14%) in the E1 experiment of the same reactor. These results indicate that haBTF is more efficient in removing H_2_S than the other two reactors. Furthermore, aBTF had a slight advantage over nBTF in both experiments (see Table 1).

**Table 1:** Biotrickling filters' performance in the E1, E2, and E3 experiments with E1 experiment in constant C_i_ at 2000 ppm and decreasing EBRT from 120 to 20s**,** E2 experiment in constant EBRT at 60s and increasing C_i_ from 1000 to 6000 ppm, and E3 experiment in decreasing EBRT from 120 to 15 s and constant Ci of 200, 1000, and 2000 ppm (only the data of 1000 ppm Ci has been shown)

Exp E1 (Constant C_i_)		Critical EC (g m^−3^ h^−1^)	EBRT (s)	H_2_S LR (g S-H_2_S m^−3^ h^−1^)	Maximum EC (g m^−3^ h^−1^)	RE (%)	EBRT (s)	H_2_S LR (g S-H_2_S m^−3^ h^−1^)
	haBTF	137.6	70	140.9	179.8	91.14	50	197.2
	nBTF	106.5	90	109.6	141	85.7	60	164.3
	aBTF	120.1	80	123.3	145	88.2	60	164.3
Exp E2 (Constant EBRT)		Critical EC (g m^−3^ h^−1^)	EBRT (s)	H_2_S LR (g S-H_2_S m^−3^ h^−1^)	Maximum EC (g m^−3^ h^−1^)	RE (%)	EBRT (s)	H_2_S LR (g S-H_2_S m^−3^ h^−1^)
	haBTF	167.8	60	173	210.6	64	60	328.7
	nBTF	137.5	60	141	150.8	76.4	60	197.2
	aBTF	144.6	60	149.1	174.3	53	60	328.7
Exp E3 (decreasing EBRT) Ci: 1000 ppm		RE (%)						
		EBRT (s):	120	80	40	20	15	
		haBTF	100	100	94	79	63	
		nBTF	100	97	75	42.5	32	
		aBTF	100	98	79	50	38	

**haBTF E1:** Critical EC that guaranteeing RE 97% of inlet H2S was determined to be 137.6 g m^−3^ h^−1^ and EBRT 70s and Loading Rate (inlet load) is 140.9 g m^−3^ h^−1^, maximum EC 179.8 g m^−3^ h^−1^, RE 91.14% and EBRT 50 s, Loading Rate is 197 g m^−3^ h^−1^ **nBTF E1:** Critical EC that guaranteeing RE 97% of inlet H2S was determined to be 106.5 g m^−3^ h^−1^ and EBRT 90s and Loading Rate is 109.6 g m^−3^ h^−1^, maximum EC 141 g m^−3^ h^−1^, RE 85.7% and EBRT 60s, Loading Rate is 164.3 g m^−3^ h^−1^ **aBTF E1:** Critical EC that guaranteeing RE 97% of inlet H2S was determined to be 120.1 g m^−3^ h^−1^ and EBRT 80s and Loading Rate is 123.3 g m^−3^ h^−1^, maximum EC 145 g m^−3^ h^−1^, RE 88.2% and EBRT 60s, Loading Rate is 164.3 g m^−3^ h^−1^

**haBTF E2:** Critical EC that guaranteeing RE 97% of inlet H2S was determined to be 167.8 g m^−3^ h^−1^ and EBRT 60s and Loading Rate is 173 g m^−3^ h^−1^, maximmum EC 210.6 g m^−3^ h^−1^, RE 64% and EBRT 60s and Loading Rate is 328.7 g m^−3^ h^−1^ **nBTF E2:** Critical EC that guaranteeing RE 97% of inlet H2S was determined to be 137.5 g m^−3^ h^−1^ and EBRT 60s and Loading Rate is 141 g m^−3^ h^−1^, maximmum EC 150.8 g m^−3^ h^−1^, RE 76.4% and EBRT 60s, Loading Rate is 197.2 g m^−3^ h^−1^ **aBTF E2:** Critical EC that guaranteeing RE 97% of inlet H2S was determined to be 144.6 g m^−3^ h^−1^ and EBRT 60s and Loading Rate is 149.1 g m^−3^ h^−1^, maximmum EC 174.3 g m^−3^ h^−1^, RE 53% and EBRT 60s, Loading Rate is 328.7 g m^−3^ h^−1^ **E3:** The reduction of RE was assessed in three Ci concentrations (only the data of 1000 ppm is shown here for more information see: Figure S1 and S2) and reducing EBRT from 120 to 15 s

In E3 experiments, the effect of reducing EBRT from 120 to 15 s on RE was investigated at three constant and independent input concentrations of 200, 1000, and 2000 ppm of hydrogen sulfide in bioreactors. The results in Figure S1 show that reducing EBRT from 120 to 80 s in haBTF at the three mentioned concentrations has no effect on the reduction of RE, and even when the inlet concentration is 1000 ppm, by reducing the time to 40 s, only 6% of RE is reduced. It has been observed that the removal efficiency reduction curves for haBTF have a lower slope compared to aBTF and nBTF, for all three concentrations. This indicates that the reduction of removal efficiency with the reduction of EBRT in haBTF is slower and independent of the inlet concentration. Based on the results obtained, it can be concluded that aBTF exhibits better performance as compared to nBTF (Table 1).

Based on the outcomes of three experimental conditions, it has been concluded that filters exposed to more stringent pH conditions perform better at a constant inlet concentration. However, when the inlet concentration varies at a constant EBRT, the result is different. In such a case, nBTF performs slightly better than aBTF.

Throughout the experiments, a minimum oxygen-to-sulfide ratio of 25 was maintained. This ensured that there was enough oxygen to completely oxidize the sulfide. Keeping sulfide at a minimum allowed for ideal conditions for complete oxidation to sulfate. This is important because sulfate provides more energy for biomass development than relative oxidation and biosulfur production. However, if the oxygen level is lower than the sulfide level, biosulfur will form and accumulate on the packing bed, causing a pressure drop and potentially blocking the system [28]. Additionally, biofilm formation on the packing media can reduce oxygen penetration, requiring more oxygen supply in aerobic biodegradation systems [18, 29].

Generally, sulfide-to-sulfate oxidation was almost complete during the experiments (more than 88% and 12% of applied sulfide converted to sulfate and biosulfur, respectively). Also, 81–90%, 75–87%, and 78–88% of H_2_S were removed at alkaline, neutral, and acidic conditions, respectively, and were assessed by measuring the outlet concentration of hydrogen sulfide placed at the first half of the column height, showing that the most of the H_2_S was removed at this part. Jia et al. Have reported almost the same rate of conversion in their acidic BTF with 89% of H_2_S converted to S^0^, but despite higher average biofilm formation in alkaline BTF than in acidic reactor, less H_2_S conversion (77%) was observed [30]. This disagreement is probably because of the mass transfer limitation which is more acute in haBTF since hydrogen sulfide (H_2_S) is a weak acid (pKa = 7), it has been observed that the pH level of the MSM solution can influence its ability to transfer mass from gas to liquid phase. However, this effect is less significant at acidic or neutral pH levels. To address this challenge, the present study conducted periodic relative starvation, which resulted in a higher conversion rate of hydrogen sulfide as compared to Jia et al.'s report. This fact is also a reason why a change in retention time will have less impact on mass transfer under alkaline conditions than under acidic or neutral pH levels.

Under conditions of constant hydrogen sulfide concentration and declining EBRT, hydrogen sulfide will transfer more mass than oxygen due to its higher concentration. In the absence of sufficient oxygen content for complete sulfide oxidation, the sulfur-to-sulfate ratio will rise [9]. The ratio of O_2_:H_2_S gradually decreases as the input pollution load increases, and with less oxygen present in the recycling medium, the oxidation of HS^−^ is incomplete, leading to an increase in the proportion of elemental sulfur in place of sulfate. On the other hand, a decrease in EBRT reduces gas-to-liquid mass transfer, while an increase in hydrogen sulfide concentration leads to an increase in biosulfur production due to the biological potential limitation in HS^−^ to SO_4_^2−^. This is the result of a decrease in oxygen content in the recycling medium and incomplete HS^−^ oxidation [31].

### Bacterial community diversity analysis

After ending all experiments (90 days), to investigate the correlation of various bacterial populations on the efficiency of H_2_S removal, an analysis of bacterial community and structure was carried out using three amplicon libraries: haBTF, nBTF, and aBTF. After deleting the wrong identified or low-quality sequences, a total of 160,632 tags (69% of initial) were identified with an average length of 424 bp, which comprised 61,264 tags in the library of haBTF, 50,216 tags in library nBTF and 49,152 tags in library aBTF. The clustering of all tags to OTU was investigated at 97% similarity and the relative abundance data was calculated for each library. A total of 290 bacterial OTUs were obtained from three amplicon libraries and 119, 134, and 37 OTUs were assigned to haBTF, nBTF, and aBTF, respectively. At the phylum level, (data has not been shown) *proteobacteria* were the most dominant phylum in all samples, so more than 70% of the total read sequences in three BTFs were classified in this taxon. The other most abundant phyla were *Bacteroidetes* (18%, nBTF) and *actinobacteria* (30%, aBTF). The relative abundances and population structures of the bacterial communities at the taxonomical class level are shown in Figure S3. In haBTF, the most abundant classes were *Alphaproteobacteria* and *Gammaproteobacteria* (20.9% and 73.7%, respectively), in nBTF *Alphaproteobacteria*, *Betaproteobacteria*, *Gammaproteobacteria* and *Flavobacteria* (3.7, 59.6%, 15% and 16.6%, respectively) were more important and in aBTF sample *Acidithiobacillia* and *Actinobacteria* (65.4% and 30.4%, respectively) were the most prevalent classes. In terms of phylogeny, *Acidithiobacillia* is classified as an independent class of in the *Proteobacteria*, but previously its type order *Acidithiobacillales* was classified in the *Gammaproteobacteria*. The *Betaproteobacteria* includes the most abundant sulfur-oxidizing bacteria, but it has been reported that in very harsh environmental conditions most SOB belongs to the *Gammaproteobacteria* [32] and in extremophile acidic conditions**,** *Acidithiobacillia* play a key role. Table S1 lists the number of OTUs predicted in the data set per taxon.

**Fig. 2:**
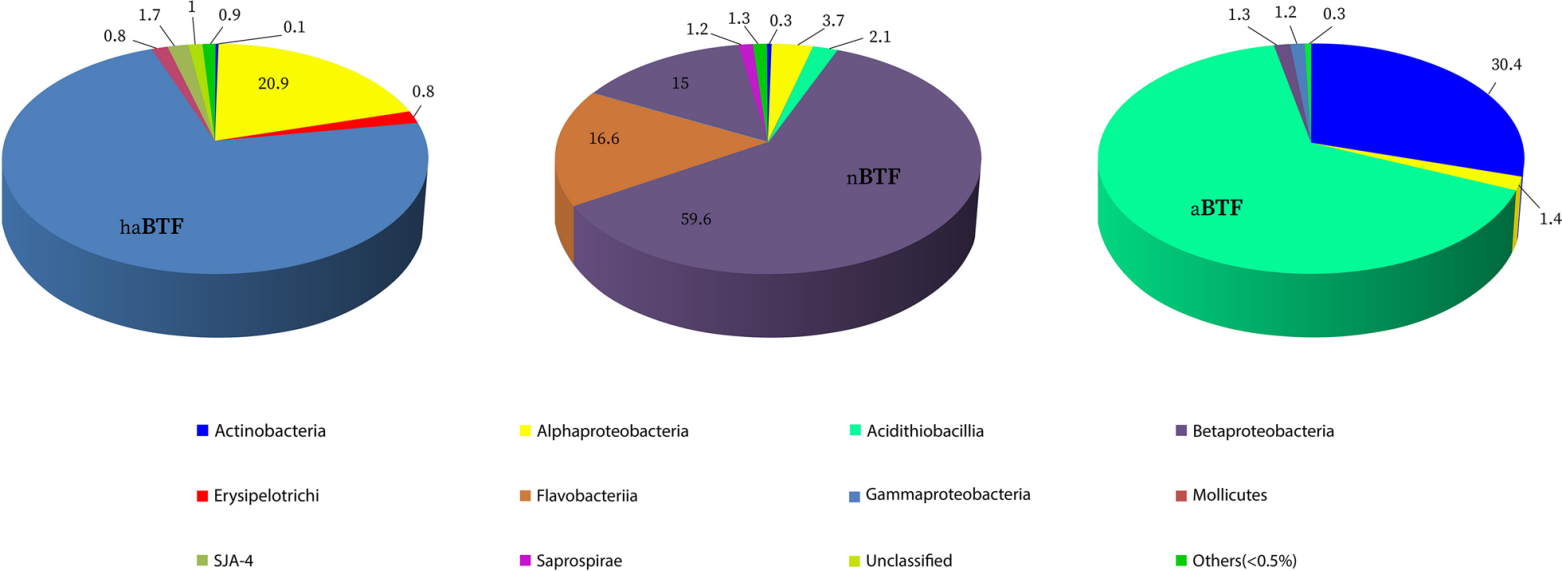
Bacterial community structure and relative abundances at a class level of haBTF, aBTF, and nBTF samples

The results of molecular studies (Fig. 2) showed that in the alkaline condition of haBTF, the *Gamma* and *Alphaproteobacteria* are the most abundant classes (73.7% and 20.9% respectively). In neutral and acidic pH conditions the frequency of these taxa decreased so the abundance of *Gammaproteobacteria* decreased to 15% and 1.15% and of *Alphaproteobacteria* to 3.7% and 1.4%, respectively. Under acidic pH conditions, the most abundant classes were *Acidithiobacillia* and *Actinobacteria* with 65.4% and 30.4%, respectively. In nBTF, the *Betaproteobacteria*, *Flavobacteria,* and *Gammaproteobacteria* were the most abundant classes, respectively.

In the present study, contrary to the results of Omri et al., [23] and Chouari et al., [24], the lowest ecological diversity was observed at acidic pH, and in accordance with the results of Tu et al. [22], the microbial diversity at neutral pH is higher than that of acidic pH. *Acidithiobacillus* was the most abundant group of bacteria in the mentioned study which is in agreement with the results of the relative abundance of different bacterial groups in three tested conditions of the present study. As shown in Table 2, *Acidithiobacillus* and *Thiobacillus* are the most abundant genera of bacteria in aBTF and nBTF, respectively and haBTF was abundant with unclassified groups of bacteria. The superiority of *Acidithiobacillus* and *Thiobacillus* has also been proved in Zhang et al.'s 2022 acidic BTF [33]. In aBTF after the *Acidithiobacillus* the most abundant acidophilic microorganism is *Mycobacterium* with an abundance of 27%, which is most likely mixotrophic SOB, and consistent with the results of Jia et al. [34], the abundance of mixotrophic SOB *Mycobacterium* was 78.4%. in aBTF with extremely acidic conditions.

**Table 2:** Phylogenetic classification up to class level and relative abundances (%) of species (Underlined genus and/or species correspond to known SOB species)

	Taxonomical affiliation		Relative abundance (%)		
Class	Family	Species	haABTF	nBTF	aBTF
*Alphaproteobacteria*	*Hyphomonadaceae*	*Oceanicaulis* sp.^2^	1.3	0	0
	*Rhodobacteraceae*	*Rhodobaca* sp.^1^	10	0	0
	*Rhizobiaceae*	*Shinella* sp.^2^	0.1	0.8	< 0.1
	*Sphingomonadaceae*	Sphingomonas^5^ sp.	0	< 0.1	< 0.1
		*Sphingomonas—wittichii*	0	0	1.3
*Betaproteobacteria*	*Hydrogenophilaceae*	Thiobacillus^3^ sp.	0	56.5	1
	-	*Thiomonas*^5^ sp.	< 0.1	2.2	0
*Gammaproteobacteria*	-	*Alkalimonas amylolytica*^4^	6.9	0	0
	-	*Alkalimonas delamerensis*^4^	1.5	0	0
	*Rhodanobacteraceae*	*Dyella*^5^ sp.	0	1.2	< 0.1
	*Halomonadaceae*	*Halomonas*^4^ sp.	1.4	0	0
		*Halomonas campisalis*	3.4	0	0
	*Halothiobacillaceae*	*Halothiobacillus*^3^ sp.	0.4	2.3	0
	*Alteromonadaceae*	*Marinobacter*^4^ sp.	1	0	0
	*Lysobacteraceae*	*Stenotrophomonas*^4^ sp.	< 0.1	0.3	0
	–	*Stenotrophomonas acidaminiphila*	< 0.1	2.8	0
	–	*Thermomonas*^4^ sp.	0	1.3	0
	*Ectothiorhodospiraceae*	*Thioalkalivibrio sulfidophilus*^3^	1.1	0	0
*Acidithiobacillia*	*Acidithiobacillaceae*	*Acidithiobacillus*^3^	0	1.3	62.5
		*A. albertensis*	< 0.1	0.8	2.9
*Actinobacteria*	*Mycobacteriaceae*	*Mycobacterium*^5^ sp.	0	< 0.1	**27**
		*Mycobacterium arupense*	0	< 0.1	3.3
*Flavobacteriia*	*Flavobacteriaceae*	*Chryseobacterium*^4^ sp.	< 0.1	14	0
*Sphingobacteriia*	*Chitinophagaceae*	*Sediminibacterium*^4^ sp.	0	0.7	0
*Mollicutes*	*Acholeplasmataceae*	*Acholeplasma*^4^ sp.	0.8	0	0
*Erysipelotrichia*	*Erysipelotrichaceae*	PSB-M-3^4^	0.8	0	0
		Total	29	84.7	98.2
		Unclassified	69.7	11.7	0.5
		Others (< 0.5%)	1.3	3.6	1.3

1. Uncultured bacterium, 2. Unclassified Bacteria, 3. Autotrophic, 4. Heterotrophic, 5. Mixotrophic

#### OTU rank curve

**Fig. 3:**
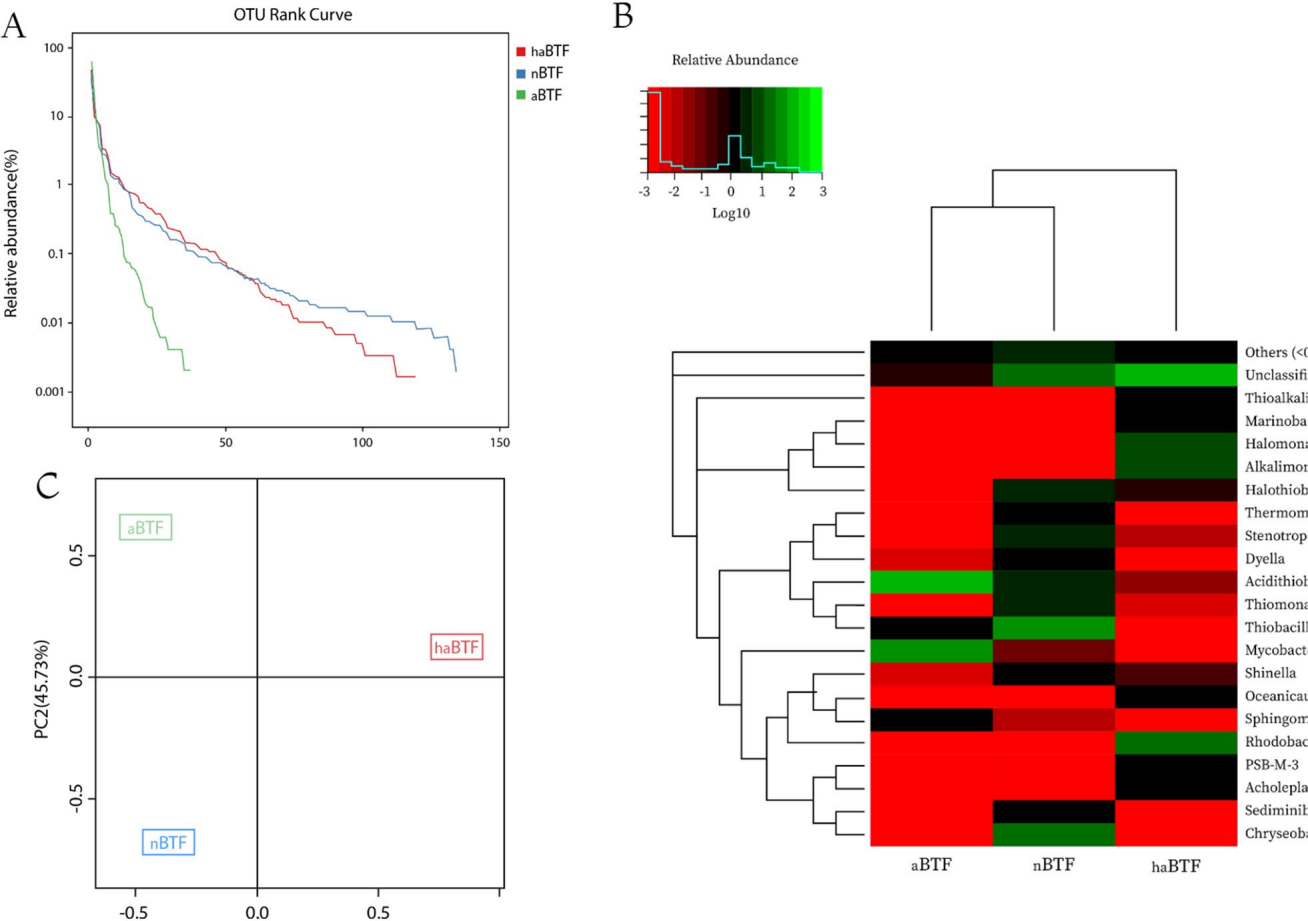
Bacterial community structure and diversity of three BTFs. **A** OTU rank curves of haBTF, nBTF, and aBTF libraries. **B** Bacterial community structures were exhibited by a heat map of abundant bacteria at the genus level. The dendrogram on the longitudinal axis was based on the sequence similarity between phylogenetic trees at the genus level and the horizontal dendrogram shows the proximity of the three bacterial communities, the relative distance was calculated based on the abundance, and the closest ones are placed in one branch and the farthest in a separate branch. The gradation of green represents the level of relative abundance as a base of log10 for all the three samples considered contigs, 0 (white) being the lowest and 7 (dark green) the abundance level of maximum considered. **C** Principal Component Analysis (PCA) based on OTU abundance

The species diversity of a population depends on two factors: species richness and evenness. Increasing species richness (X-axis) simultaneously with uniform distribution of abundances (Y-axis log scale) can dramatically increase species diversity. OTU rank abundance curve can visually depict two factors at the same time. As can be seen in Fig. 3A in terms of species richness, nBTF has the highest and aBTF has the lowest richness. The steep slope of the OUT curve indicates the lack of uniform distribution of abundance in aBTF so that a limited number of OUTs ranks have the highest frequency and more numbers have the lowest frequency. The curves of haBTF and nBTF, at first start with steep slopes and then vary gently downward parallel, so the two BTFs are similarly distributed in terms of species evenness, and both have more evenness than the aBTF. Overall, according to OTU rank curves, haBTF and nBTF have the same species diversity and more than the aBTF.

#### Rarefaction curves

Based on observed OTUs with increasing sequencing, it was shown that the three rarefaction curves tend to be smooth. This suggests that the produced data is enough to cover the diversity of all species in the community. The other rarefaction curves based on the Chao1 and ACE values also confirmed these results (Figure S4).

#### Heat map analysis

Heat map analysis was done based on the relative abundance of each genus in each sample. To minimize the differences degree of the relative abundance value, the values were all log-transformed. The gradation of green shows the relative abundance of the three samples, −3 (light red) being the minimum and 3 (light green) the abundance of maximum considered. As shown in the heat map (Fig. 3B), haBTF contained a more diverse group of bacteria in comparison to the two other reactors. This may be because of the formation of a pH gradient in haBTF which provides an adverse niche for different genera. The pH gradient may also be formed in nBTF but in less adverse value than that of haBTF. The most abundant known genera in aBTF, nBTF, and haBTF were *Acidithiobacillus*, *Thiobacillus,* and *Rhodobaca*, respectively. The effect of pH on the formation of different microbial communities has been reported in previous reports by Chouari et al., [24] and Tu et al., [22] in which they corroborated changing pH from neutral to acidic conditions will cause alteration in microbial population and both insisted on attendance and abundance of *Acidithiobacillus* in (extremely) acidic biofilters.

#### Alpha diversity

According to results in Table 3, the value of observed species and indexes of Chao1and ACE showed species richness at neutral pH of nBTF is considerably higher than the others, and also the species richness at the haloalkaline condition (pH 8*.*5) is higher than the acidic pH (2–3.5). Severe acidic conditions as selective pressure decreased species richness, but despite the relatively high salt concentration and slight alkalinity in haBTF, seemingly these limiting factors had less effect on species richness.

**Table 3:** Diversity indexes. Observed species (Sobs) value, chao1, ACE indexes reflect the species richness of the community, Shannon and Simpson indexes reflect species diversity

Sample Name	Sobs	Chao1	ACE	Shannon	Simpson
haBTF	119	121.3	122.2	2.35	0.23
nBTF	134	134	134.1	2.26	0.23
aBTF	37	37.4	39	1.25	0.44

Shannon value (H) and Simpson index (D) estimators can reflect the diversity (richness and evenness species) of the community. Shannon value is influenced more by rare abundant species while the Simpson index (D) is affected by dominant and even species. According to the results of Shannon's value, the community diversity of haBTF is greater than the other samples and aBTF is in the lowest level of diversity, but Simpson's index indicated species diversities of haBTF and nBTF are the same and they are greater than aBTF due to its much less evenness and richness.

#### OTU PCA analysis

To display the differences in OTU composition in three BTF samples, Principal Component Analysis (PCA) was used to construct a 2-D graph to summarize factors mainly responsible for this difference. Figure 3C, based on principal component analysis, showed that along the PC1 axis, the samples could be divided into two separate groups, and along the PC2 axis, they could be separated into two other groups. The composition of OTUs in haBTF is significantly different from that of aBTF and nBTF, and according to the distribution of aBTF and nBTF along the vertical axis of PC2, it could be concluded that these two populations are more similar in terms of OTU structure. These results showed that the bacterial populations of haBTF have a significant difference compared to the other two BTFs, and on the other hand, the bacterial populations formed in aBTF and nBTF are more similar to each other.

### Scanning electron microscopy

**Fig. 4:**
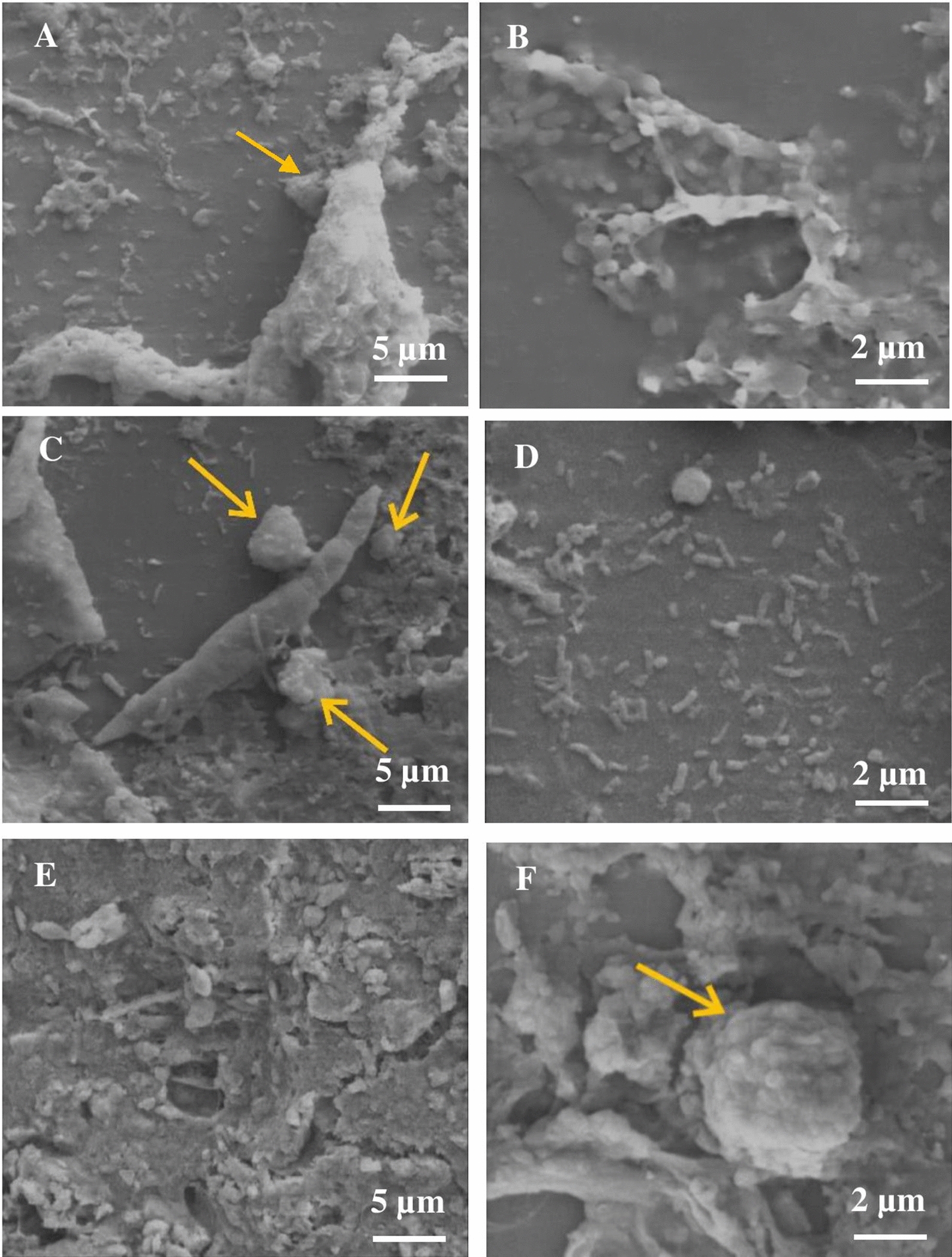
Scanning Electron Microscopy images of the packing material after the desulfurizing operation. (haBTF: **A** X2000, **B** X5000), (nBTF: **C** X2000, **D**. X3000), (aBTF:** E** X1000, **F** X3000). Yellow arrows point biosulfur precipitants

Scanning electron micrographs were utilized to track the development of biofilm on pallrings after 90 days of experiments (Fig. 4). The analyses revealed the presence of a complex community of bacteria in the haBTF samples (Fig. 4A and B), including filamentous bacilli that were 1.5 μm in diameter and 4 μm in length, rod-shaped bacteria that were 0.4 μm in diameter and 1 μm in length, and cocobacilli that were 0.5 μm in diameter and 1 μm in length. In addition to bacterial aggregation, biosulfur sediments are shown in Fig. 4B. In nBTF samples (Fig. 4C, D), there are bacteria with diameters ranging from 0.34 to 0.9 μm and lengths ranging from 0.9 to 9 μm, biosulfur accumulations produced by SOB secretions with diameters ranging from 1.4 to 7 μm (pointed with yellow arrows), and a nematode (nearly 5 μm in diameter and 35 μm in length), in addition to various sized bacteria. Even though the experiment used lethal levels of hydrogen sulfide, the existence of nematodes and other protozoa in neutral pH biofilters was unexpected. Amorphous fouling and spherical accumulations of biosulfur (diameter 8–10 μm) were also seen in aBTF-associated biofilm (Fig. 4E, F)).

Upon comparing the images of the three filters, it is evident that haBTF (Fig. 4B) has a thicker and more condensed biofilm layer in which rod-shaped microbial strains were trapped in large amounts of EPS as compared to the acidic and neutral ones. This observation is consistent with the findings of Jia et al., who reported a higher rate of biofilm formation in alkaline BTF than in acidic conditions [30]. It is worth noting that nematodes were only observed in the neutral biofilter. Although this was unexpected due to the presence of lithic concentrations of H_2_S, it could indicate successful pH control during the experiments. This group of organisms are predators of bacteria and therefore play a vital role in controlling their population [35].

## Conclusions

Assessing the effect of pH conditions (haloalkaliphilic, neutrophilic, and acidophilic) on the efficiency of H_2_S removal in the BTF system, three parallel experiments were performed in which removal-determining parameters showed 91.14% RE and 179.8 gS-H_2_S m^−1^ EC in haBTF when EBRT was 60 s. These values differed in nBTF and aBTF with 85.7% and 88.2% RE; 141 and 145 gS-H_2_S m^−1^ EC, respectively. These results besides a lower reduction slope of RE (%) in haBTF with a decrease in EBRT compared to nBTF and aBTF demonstrated better performance of haloalkaliphilic BTF in H_2_S removal. This biofilter had also distinct bacterial diversity in comparison with nBTF and aBTF, as NGS results showed, and its biofilm structure seemed more condensed with higher EPS content based on its SEM images. Despite less bacterial diversity, acidic conditions performed slightly better compared to the neutral system (see Table 4) at a constant inlet concentration, revealing that biotrickling systems with harsh pH conditions perform better in removing H_2_S than neutral filters.

**Table 4:** Comparison of the operation and microbial diversity of haBTF, nBTF, and aBTF

	haBTF	nBTF	aBTF
RE in constant Ci (%)	91.14	85.7	88.2
EC_max_ in constant Ci	179.8	141	145
RE in constant EBRT (%)	64	76.4	53
EC_max_ in constant EBRT	210.6	150.8	174.3
RE reduction in decreasing EBRT (%)^1^	37	68	62
The most abundant classes	*Alphaproteobacteria* *Gammaproteobacteria*	*Betaproteobacteria*	*Acidithiobacillia*
The most abundant families	*Rhodobacteraceae*	*Hydrogenophilaceae*	*Acidithiobacillaceae*
The most abundant species	*Alkalimonas amylolytica* *Halomonas campisalis*	*Thiobacillus*^*3*^ sp.	*Acidithiobacillus sp.*
Alpha diversity^2^	2.35	2.26	1.25
Biofilm thickness^3^	+ + +	+	+ +

1 Constant Ci: 1000 ppm, 2 Based on the Shannon index, 3 Based on the SEM results (qualitative)

## Materials and methods

### Culture media composition

The nutrient solutions of recirculating and enrichment cultures were the mineral salt media (MSM) (Table S2). MSM_ha_, MSM_n,_ and MSM_a_ were used in haloalkaline biotrickling filter (haBTF), neutral biotrickling filter (nBTF), and acidic biotrickling filter (aBTF), respectively. All chemicals were of analytical grade with more than 99.5% purity and were purchased from Merck.

During the enrichment and start-up periods, the sole sulfur source for the SOB growth was supplied by Na_2_S_2_O_3_$\cdot$5H_2_O solution (10 g L^−1^), and H_2_S mix gas was used during the acclimatization and the experimental phases.

### Enrichment, acclimatization and immobilization of SOB consortia

For acquiring diverse and active microbial sulfur-oxidizing consortia as initial inoculum, the SOB community was enriched from a mixture of the following materials: activated sludge of urban and industrial wastewater treatment plants (petrochemical and leather processing industries). At the beginning of the enrichment process, 350 g of the mixture as mentioned earlier was mixed with 2 L of MSM_n_ and MSM_ha_ culture media individually and supplemented with Na_2_S_2_O_3_$\cdot$5H_2_O (10 g L^−1^) as the sole energy source. Enrichment cultures were continuously aerated for about 6 weeks. The pH values of MSM_n_, MSM_ha_, and MSM_a_ were automatically controlled at 7, 8.5, and 1.5 to 2 respectively using 0.5 M NaHCO_3_ (for MSM_ha_) or 2N NaOH (for MSM_a_ and MSM_n_) when necessary and the temperature was controlled at 30 °C. At this phase, 30% of the culture media was replaced by fresh media every week and the process continued for 6 weeks until the density of the enriched microbial consortium increased to 10^7^–10^8^ CFU ml ml^−1^. At the immobilization period (startup period), BTFs were inoculated with 500 ml of enriched media harboring 10^7^–10^8^ CFU ml^−1^ of bacteria. Sodium thiosulfate was replaced by H_2_S as the sole source of energy and to allow the development of biofilm on the packing media, H_2_S concentration was gradually increased stepwise from 100 to 2000 ppm over 62 days with an empty bed residence of 120s [35–39].

### Biotrickling filter setup

**Fig. 5:**
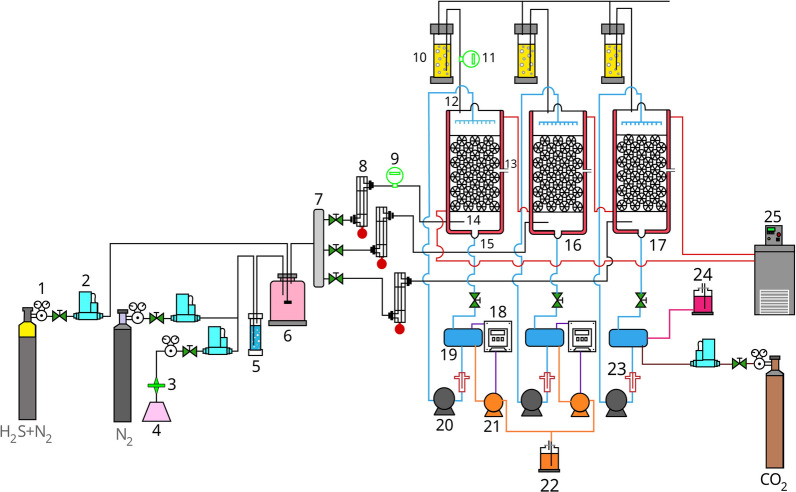
Schematic of BTFs system. 1. Gas regulator; 2. Mass flow controller; 3. Filter; 4. Compressed air; 5. Humidifier; 6. Gas mixing chamber; 7. Gas distributor; 8. Soap bubble flowmeter; 9. Inlet H_2_S gas sensor; 10. NaOH traps; 11. Outlet H_2_S gas sensor; 12. Gas outlet; 13. Middle sampling port; 14. Gas inlet; 15. haBTF; 16. nBTF; 17. aBTF; 18. pH controller; 19. MSM reservoir; 20. Recirculation pumps; 21. pH controler pump; 22. NaOH reserve; 23. Biosulfur gravity drain trap; 24. NaHCo_3_ reserve; 25. Water bath circulator

As shown in the schematic drawing of the experimental setup (Fig. 5), three identical lab-scale BTFs were operated to study the performance of H_2_S removal under haloalkaline, neutral, and acidic conditions. The BTF glass columns had an internal diameter and a height of 8 and 60 cm respectively and were packed with polypropylene 16 mm Pall rings to the height of 50 cm (Pall Ring Company, UK). The column temperature was controlled at 30°C by continuous water circulation in the external jacket (Lauda RC6-CS, Germany). There were three sampling ports in the inlet, outlet, and middle sections of the column. In each BTF, the MSM was fed and recirculated over the packing media by using a peristaltic pump (Watson-Marlow Inc WM603s, USA) through a spray nozzle located at the top of BTFs. The trickling liquid velocity (TLV) During the startup stage was set to 0.9 m h^−1^ and during all experiments was set to 7 m h^−1^.

Before recycling culture media to the reactor, the coarse sulfur particles were separated by gravity sedimentation in 200 ml sludge traps. To prevent any release of H_2_S gas, the outlet gas of BTFs passed through the NaOH trap. The pressure drops of reactors (∆P) due to biofilm growth were monitored with U-tube water manometers connected to the bottom and top sections of the columns. The air supplied by a compressor was filtered and passed through a thermal mass flow controller (MFC) (BROOKS Instruments USA, type 6250s). Nitrogen flow gas (N_2_, 99.995%) was controlled by Digital MFC) BRONKHORST Instruments Netherlands, type EL-FLOW-201CV. Air and nitrogen were humidified by passing through a water bubble column.

A cylinder of H_2_S gas (5%, balanced N_2_) was used for supplying hydrogen sulfide and a Digital MFC (UNIT Instruments USA, type UFC-1661) was used to control the flow of gas. Synthetic polluted air was created by combining H_2_S with humidified N_2_ and air mixture, in a mixing chamber and divided equally into three flows using a three-way distributer, the mixed gas flow rate was controlled by rotameters before entering each reactor. The gas current was supplied from the bottom of BTFs through a diffuser and the treated gas discharged from the upper end of the column. During the operation times, to prevent the toxic effects of by-products and supply fresh nutrient sources, 15% of spent liquid media was replaced with fresh MSM daily. The pH of MSM_n_ and MSM_a_ in the nBTF and aBTF were monitored and controlled at set-points of 7 ± 0.1 and 2.5 ± 0.5 respectively by automatic addition of 2 N NaOH by pH controller and dosing pump (Hanna BL 7916, USA). The pH and alkalinity of MSM_ha_ in haBTF were adjusted at a value of 8.5 ± 0.1 and 0.4 M, respectively using the addition of make-up fresh medium. If the pH of the MSM_ha_ was higher than 8.5, carbon dioxide gas was used to adjust the pH. Compensation for evaporated water in bioreactors was done by adding water weekly. In all designed experiments, the amount of circulating dissolved oxygen (DO) was considered to be higher than the requirement for complete sulfide oxidation. If the DO of the recirculating medium was reduced compared to the saturated state, excess oxygen was purged to the recirculation medium in the MSM reservoir.

To control filters' clogging during operation, a new method of periodic relative starvation was used. This method is based on the observation that the growth of biofilm is higher near the inlet due to the higher concentration of the substrate, making it more prone to clogging and blockage. In such cases, the U-shaped manometer shows the pressure drop. So, to prevent clogging and maintain the activity of the column, the position of the polluted air inlet was changed alternately by moving the input along the length of the column at different time intervals. This allows the substrate to be spread and a biofilm to be formed along the entire length of the column, increasing the length of time the column operates without the need for washing to remove biofilm and sulfur from the bed.

### Experimental conditions and methodology

After 62 days of establishing stable conditions in all three bioreactors (E1, E2, and E3), experiments were conducted to evaluate and compare the performance of BTFs. The conditions of conducting these experiments are shown in Table 5. Experiments E1 and E2 were conducted in two periods of 11 days each, while experiment E3 was conducted in a period of 15 days. To evaluate system performance, parameters such as load retention (LR), elimination capacity (EC), and removal efficiency (RE) were calculated by measuring the inlet (C_i_) and outlet (C_out_) concentrations and using retention time and flow rate (Q). By decreasing the empty bed residence time (EBRT) while keeping (C_i_) constant or by increasing (C_i_) and keeping EBRT constant, the contamination load of bioreactors can be increased, and the behavior of BTFs can be studied. (see the Eq. (1)) [40]:

1 $${\text{LR }} = {\text{ Q}}.{\text{C}}_{{\text{i}}} /{\text{ V}}_{{\text{b}}} = {\text{ C}}_{{\text{i}}} /{\text{ EBRT,}}$$

where LR is load retention (gS m^−3^ h^−1^), C_i_ is inlet concentration (gS m^−3^), Q is the flow rate (m^3^ h^−1^), V_b_ is the volume of the packed bed (m^3^), and EBRT is empty bed retention time (h).

**Table 5:** Experimental conditions for the biotrickling filters (haBTF, nBTF, aBTF)

Experiment	C_i_ (ppm)	LR (gS-H_2_S m^−3^ h^−1^)	EBRT (s)	Duration (d)	Previous operation (d)
E1	2000	82.19	120	2	31
		89.16	110	2	
		98.63	100	2	
		109.59	90	2	
		123.29	80	2	
		140.90	70	2	
		164.38	60	2	
		197.26	50	2	
		246.58	40	2	
		328.77	30	2	
		493.16	20	2	
E2	1000	82.19	20	2	53
	1090	89.16	20	2	
	1200	98.63	20	2	
	1333	109.59	20	2	
	1500	123.29	20	2	
	1714	140.90	20	2	
	2000	164.38	20	2	
	2400	197.26	20	2	
	3000	246.58	20	2	
	4000	328.77	20	2	
	6000	493.16	20	2	
E3	200	65.76	15	1	75
		49.32	20	1	
		24.66	40	1	
		12.33	80	1	
		8.22	120	1	
	1000	328.8	15	1	80
		246.6	20	1	
		123.3	40	1	
		61.65	80	1	
		41.1	120	1	
	2000	665.76	15	1	85
		499.32	20	1	
		249.66	40	1	
		124.83	80	1	
		83.22	120	1	

#### The effects of EBRT on BTF's operation

During the 22-day E1 experiments, the EBRT was gradually reduced from 120 to 20 s while maintaining a constant C_i_ of 2000 ppm. This resulted in an increase in LR from 82 to 493 g m^−3^ h^−1^. To ensure performance stability, each period was maintained for 48 h. Table 5 displays the details of the E1 experiments' conditions. At the end of each stage of EBRT change, inlet and outlet hydrogen sulfide concentrations were measured, and EC and RE values were calculated. Critical parameters such as EC_critical_ and EBRT_critical_ were determined at the points where the RE reached 97% [41]. The calculation of maximum EC and critical EBRT can provide valuable information for bioreactor modeling, performance, and design. The third experiment (E3) was conducted in three C_i_ concentrations (200, 1000, and 2000 ppm) to assess the impact of decreasing EBRT (from 120 to 15s) on removal efficiency (RE). See Table 5 for further experiment conditions.

### The effects of inlet concentration on BTF's operation

According to the results obtained in the E1 experiments, in E2 experiments, EBRT was kept constant at 60 s, and C_i_ was gradually increased from 1000 to 6000 ppm in 22 days (LR 82 to 493 g m^−3^ h^−1^). The design of BTFs can benefit from the reduction of EBRT which can lead to a reduction in the height of the column and the initial construction costs. To ensure stable performance, each period of increased LR was continued for 48 h in E1 and E2, and 24 h in E3, refer to Table 5 for detailed test conditions.

### Microbial diversity analysis

#### DNA extraction

At the end of experiments, to evaluate the changes in microbial communities as a function of the BTFs pH values, 30 g of packed bed with attached biomass was collected from top, middle, and bottom sections along the length of the reactors in aseptic condition. Total Genomic DNA was extracted and purified from biomass, using Fast DNA^®^ Spin Kit for Soil (MP Biomedicals, USA) following the manufacturer's instructions, but the binding time of DNA to a silica matrix was modified to 20 min. For each sample, all the above-mentioned operations were performed in triplicate. The quantity and quality of the extracted DNA were analyzed by NanoDrop UV–VIS Spectrophotometer (Thermo Fisher Scientific, NanoDrop ONE, USA). Structural DNA integrity was evaluated by gel electrophoresis analysis (concentration of agarose gel 1%, voltage 150 V, electrophoresis time 40 min).

#### NGS analysis

DNA samples were sent to Beijing Genomics Institute (BGI, Hong Kong NGS Lab) for bacterial diversity analysis based on BGI protocols. Briefly, DNA sample quality control was done by Qubit Fluorometer, NanoDrop, and gel electrophoresis. Then, three libraries of the V3–V4 hypervariable regions (approximately ~ 460 bp) of 16S rRNA genes amplicon were constructed and qualified. Libraries were pair-end sequenced on a MiSeq System (Illumina, San Diego, CA, USA) using the PE300 (PE301 + 8 + 8 + 301) sequencing strategy. The primers used in the PCR reaction were 341 F(5′-ACTCCTACGGGAGGCAGCAG-3′) and 806 R (5′-GGACTACHVG GGTWTCTAA T-3′) (Report of BGI, Hong Kong, NGS lab).

#### High-throughput sequence data analysis

Bioinformatic data analysis was conducted by the following software. Paired-end reads with sequencing adapters, N base, poly base, low quality, etc. were filtered out then clean paired-end reads were overlapped and combined to generate the consensus sequences (tags) by FLASH (V1.2.11). The tags were clustered to Operational Taxonomic Unit (OTU) with 97% pairwise identity by UPARSE-OTU algorithm in USEARCH (v7.0.1090). To calculate the abundance of each OTU, the USEARCH global method was used and the representative OTU sequence was selected according to the most abundant sequence of each OTU. Operational taxonomic units representative sequences were taxonomically classified using Ribosomal Database Project (RDP) Classifier v.2.2 trained on the Greengenes database, using 0.8 confidence values as the cutoff.

To analyze and compare the diversity and structure of bacterial communities, alpha diversity indices (Observed species, Ace, Chao1, Shannon, and Simpson) were calculated by Mothur (V1.31.2), and beta-diversity (Principal Component Analysis) was studied using the package ade4 of software R (V3.1.1). OTU's rank curve, rarefaction curves, alpha diversity indexes, and OTU's heat map analysis were done by software R (V3.1.1).

### Electron microscopy analysis

Scanning electron microscopy (SEM) images were used for the identification of microbial biofilm formation on the Pall Rings surfaces. The samples were taken from different heights of the BTF column and submerged in ringer solution for removal of planktonic cells. Then samples were fixed for 2 h in an aqueous solution of 2% osmium tetroxide and washed with water and subsequently postfixed with a solution of 2.5% glutaraldehyde in phosphate buffer for 1.5 h at 4 °C and washed in the water again. The graded series of Ethanol solutions (25, 50, 75, 100%) were used for dehydration and finally, the samples were freeze-dried for 1 day. For SEM **(**ZEISS 960A, Germany) examination, replicas were produced by shadowing with a layer of gold in a sputter coater (Balzers SCD 004, Lichtenstein) [42].

### Analytical methods

H_2_S concentration was monitored online in the sampling port at the inlet, outlet, and middle sections of reactors using electrochemical and photoionization detectors (PID). A portable electrochemical sensor (SP2nd SI-100 SENKO, Korea) was used to measure H_2_S concentration from 0 to 100 ppm, and at a concentration from 100 to 4000 ppm, a fixed TVOC system contained PID detector (Ion Science, UK) was used. The precision of online sensor measurement was confirmed monthly by gas chromatographic (GC) analysis. The GC instrument was an Agilent Technologies (model 6890N Network GC System, USA) equipped with a flame photometric detector (FPD) and HP-1 capillary column (0.25Ò 3000 mm; Hewlett Packard, USA). The initial, mild, and final oven temperature was 50, 120, and 250 °C, with temperature ramping 5 °C min^–1^ and 20 °C min^–1^. Nitrogen was used as the carrier gas at a flow rate of 1.5 ml min^−1^ and the temperature in the oven, injector, and detector were fixed at 250 °C, 100 °C, and 250 °C, respectively.

The concentration of sulfide and sulfate were measured using ion chromatography (Waters, USA) equipped with an IC-Pak anion HC column (4.6 4.6 × 150 mm; Waters) and a conductivity detector (Waters 432). Eluent consisted of 1.7 mM sodium carbonate and 1.8 mM sodium bicarbonate solution with a flow rate of 0.9 ml min^−1^. Dissolved oxygen (DO) and pH were monitored online in a liquid medium by (HQ40d, HACH) and (HANNA HI98191, USA), respectively. At the enrichment phase for the enumeration of total culturable chemolithotrophic SOB, samples were taken from the liquid medium, and the standard plate count method was used, dilutions of suspensions were then plated in appropriate MSM agar medium which was supplemented by Na_2_S_2_O_3_$\cdot$5H_2_O 10 g L^−1^ (Table S2). The plates were incubated at 30 °C and the colonies were counted after 1 month.

### Supplementary Information

Additional file1

## Data Availability

The datasets generated during and/or analysed during the current study are available from the corresponding author on reasonable request.
