# Supplementary material for: Assessing the efficiency and microbial diversity of H2S-removing biotrickling filters at various pH conditions
Source: Microb Cell Fact. 2024 May 28;23:157. doi: 10.1186/s12934-024-02427-9 (PMC11134876; doi:10.1186/s12934-024-02427-9)
Supplement: Supplementary file 1 — Additional file1 [file 12934_2024_2427_MOESM1_ESM.docx]

**Table S1.** Estimation for number of OUT per each taxon in data set

|  | Phylum | Class | Order | Family | Genus |
| --- | --- | --- | --- | --- | --- |
| haBTF | 15 | 9 | 15 | 17 | 14 |
| nBTF | 14 | 8 | 16 | 14 | 11 |
| aBTF | 5 | 5 | 10 | 8 | 6 |

**Table S2.** Composition of different mineral salt media (MSM)

|  | ^a^MSM_ha_ | | | ^b^MSM_n_ | | ^c^MSMa | |
| --- | --- | --- | --- | --- | --- | --- | --- |
| Composition (g L^-1^) | Na_2_CO_3_.10H_2_O | | 20 | Na_2_CO_3_ | 0.40 | CaCl_2_.2H_2_O | 0.25 |
|  | NaHCO_3_ | 10 | | KH_2_PO_4_ | 2.0 | KH_2_PO_4_ | 3.0 |
|  | KNO3 | 0.5 | | K_2_HPO_4_ | 2.0 | KNO3 | 1.0 |
|  | NaCl | 5.0 | | NH_4_C1 | 0.40 | (NH_4_)_2_SO_4_ | 3.0 |
|  | K_2_HPO_4_ | 1.0 | | - | - | - | - |
|  | MgCl_2_.6H_2_O | 0.10 | | MgC1_2_.6H_2_O | 0.20 | MgSO_4_.7H_2_O | 0.50 |
|  | Na_2_S_2_O_3_.5H_2_O | 10 | | Na_2_S_2_O_3_.5H_2_O | 5 | Na_2_S_2_O_3_.5H_2_O | 5 |
| Vitamine Solution (ml) | - |  | | 3 |  | - |  |
| Trace elements solution (ml) | 2 | | | 1 | | - | |
| pH | 8.5-9 | | | 7 | | 2-3.5 | |
| C:N:P:S | 60:1:2:40 | | | 1:1:10:25 | | ^d^ | |

1. According DSMZ Medium 925:

<https://www.dsmz.de/microorganisms/medium/pdf/DSMZ_Medium925.pdf>

1. According DSMZ Medium 486:

<https://www.dsmz.de/microorganisms/medium/pdf/DSMZ_Medium486.pdf>

1. According DSMZ Medium 71:

<https://www.dsmz.de/microorganisms/medium/pdf/DSMZ_Medium71.pdf>

1. Note: Examining the method of autotrophy in the acidophilic genus *Thiobacillus* shows that this bacteria can fix carbon dioxide through the Calvin–Benson–Bassham (CBB) cycle, so no additional carbon source is used in an acidic MSM mineral environment [1,2].

**
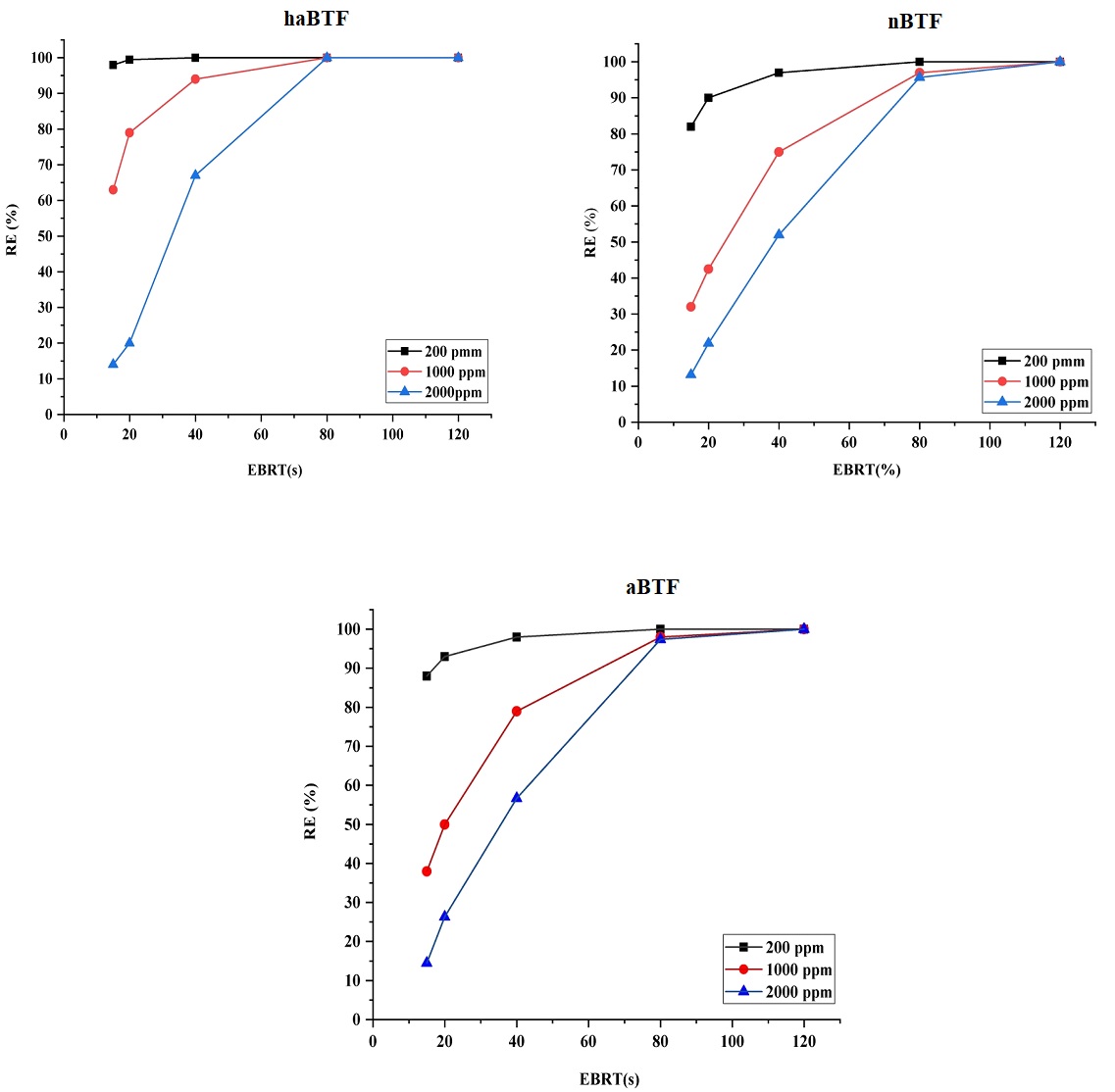
**

**Figure S1.** Effect of EBRT on the H2S removal efficiency. haBTF (A), nBTF (B), aBTF (C)


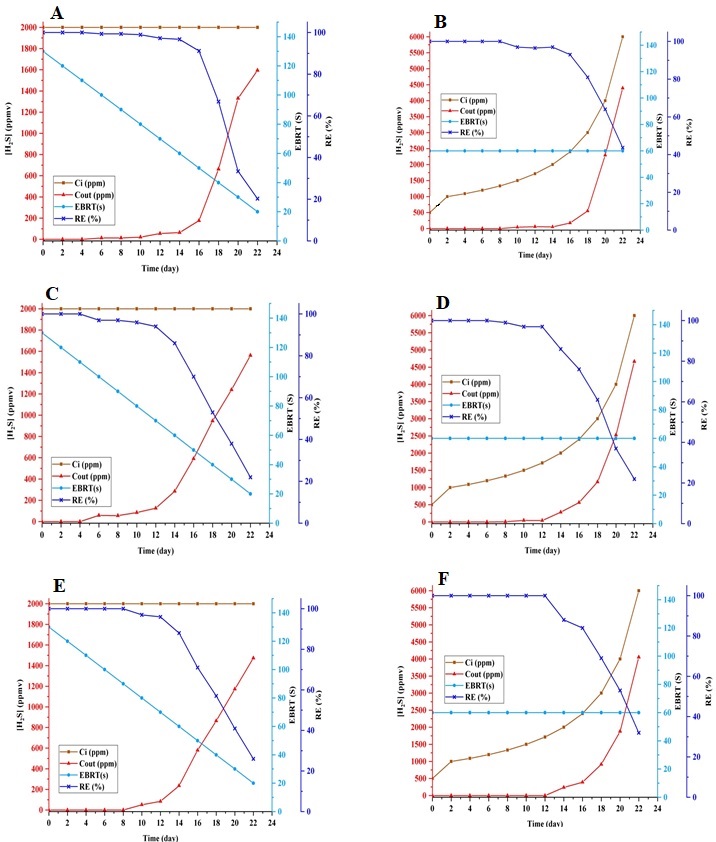


**Figure S2**. haBTF (A & B), nBTF (C & D), aBTF (E & F). (A, C, E) Effect of EBRT changes (from 120 seconds to 20 seconds) on RE and C_out_ while C_i_ remains constant. (B, D, F) Effect of increasing inlet concentration (from 1000 ppm to 6000 ppm) on RE and C_out_ while EBRT remains constant

**
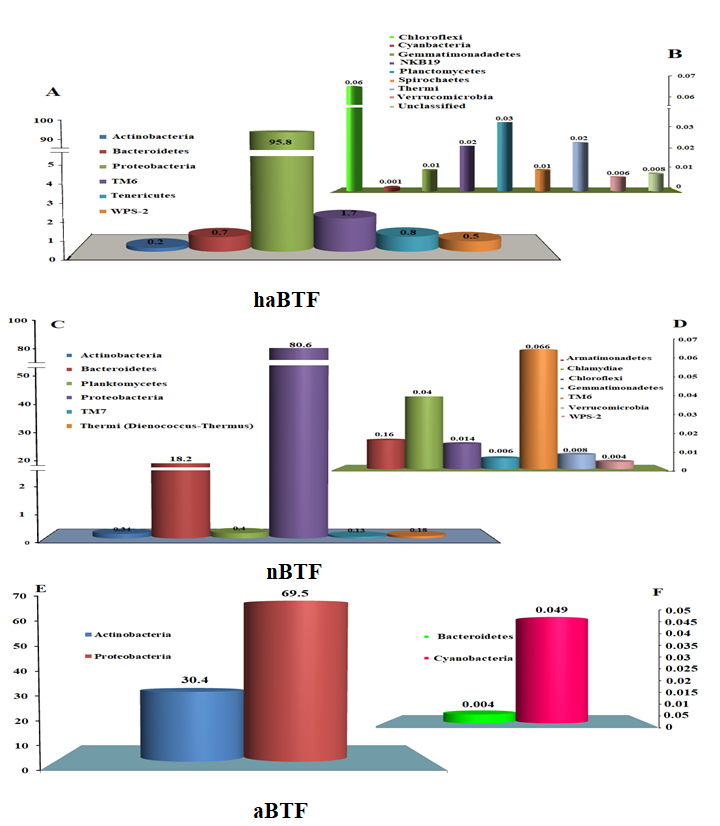
**

Figure S3. Bacterial community structure and relative abundances at a phylum level of haBTF, aBTF, nBTF samples

**
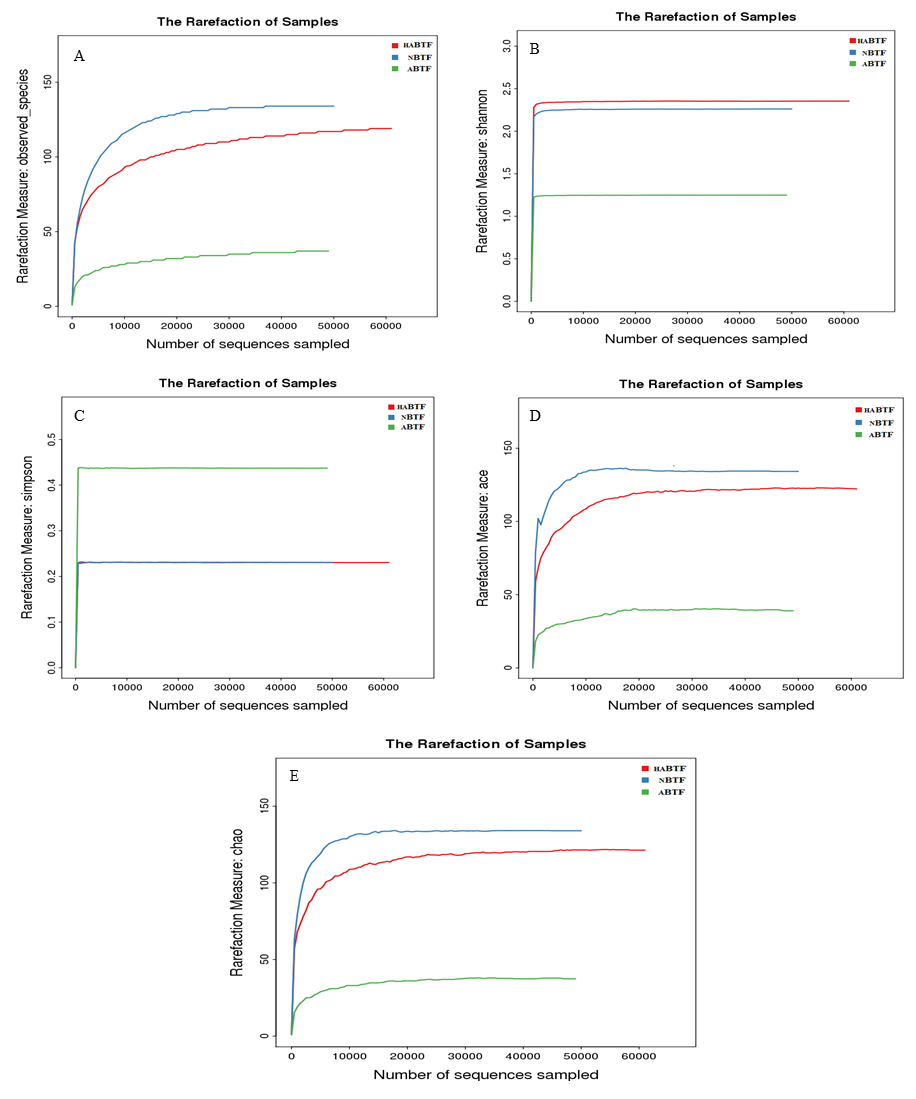
Figure S4**. Rarefaction curve for three BTFs libraries

**References**

1. Abhishek Gupta, Jayeeta Sarkar, Pinaki Sar, Chapter 33 - Understanding the Structure and Function of Extreme Microbiome Through Genomics: Scope and Challenges, Editor(s): Surajit Das, Hirak Ranjan Dash, Academic Press, 2019, Pages 581-610, ISBN 9780128148495, https://doi.org/10.1016/B978-0-12-814849-5.00033-2.
2. Noel R. Krieg, Penelope J. Padgett, Chapter 3 - Phenotypic and Physiological Characterization Methods, Editor(s): Fred Rainey, Aharon Oren,Methods in Microbiology, Academic Press, Volume 38, 2011, Pages 15-60, ISSN 0580-9517, ISBN 9780123877307, https://doi.org/10.1016/B978-0-12-387730-7.00003-6.
